# Comparison of montage with conventional stereoscopic seven-field photographs for assessment of ETDRS diabetic retinopathy severity

**DOI:** 10.1186/s40942-019-0201-z

**Published:** 2019-12-13

**Authors:** Nam V. Nguyen, Erin M. Vigil, Muhammad Hassan, Muhammad S. Halim, Sean C. Baluyot, Hugo A. Guzman, Rubbia Afridi, Diana V. Do, Yasir J. Sepah

**Affiliations:** 1 Byers Eye Institute, Stanford University, 2370 Watson Court, Suite 200, Palo Alto, CA USA; 2 Ocular Imaging Research and Reading Center, Sunnyvale, CA USA; 3 University of Texas Southwestern School of Medicine, Dallas, TX USA; 4 College of Arts and Sciences, University of Nebraska-Lincoln, Lincoln, NE USA

**Keywords:** Diabetic retinopathy severity score, Stereoscopic seven-field, Montage

## Abstract

**Background:**

The ETDRS stereoscopic seven-field (7F) has been a standard imaging and grading protocol for assessment of diabetic retinopathy (DR) severity score in many clinical trials. To the best of our knowledge, the comparison between montage and stereoscopic 7F has not been reported in the literature. Therefore, the main purpose of this study is to compare agreement between montage and stereoscopic seven-field (7F) photographs in the assessment of DR severity.

**Methods:**

Stereoscopic 7F photographs were captured from subjects with DR. Montages of monoscopic 7F images were created using Adobe Photoshop CS6 Extended©. The best quality image of each stereo pair was selected and placed on a 150 × 125-inch canvas field according to the standard location from field 1 to 7. All the fields were aligned following the vessels and overlaid using the built-in blending tool. The resulting montage was utilized for grading and compared with grading on stereoscopic 7F photographs. Three independent graders were asked to assess DR severity on stereoscopic 7F photographs and montage. Severity level agreement between stereo 7F and montage was cross-tabulated and the agreement of DR severity levels between stereoscopic 7-field images and montage was analyzed using κ intergrader agreement; statistical significance was set at p < 0.05.

**Results:**

A total of 50 eyes were included in the study. There was a substantial agreement between stereoscopic 7F and montage (κ = 0.745, κ_weighted_ = 0.867) in assessment of DR severity. Of 50 eyes, 80% of the cases showed complete agreement, and 100% of the cases had agreement within one-step. There was a moderate agreement among graders, and κ-value ranged from 0.4705 to 0.5803.

**Conclusion:**

In this study, we found a substantial agreement in assessing DR severity score employing non-stereoscopic montage and stereoscopic 7F photographs.

## Background

Diabetic retinopathy (DR), an ocular complication of diabetes, is the leading cause of irreversible blindness among Americans from age 20 to 74 years and accounts for 12% of all cases of blindness [1–3]. Patients with DR commonly present with associated vision threatening complications such as diabetic macular edema and neovascularization, which can lead to vitreous hemorrhage and retinal detachment [4]. The probability of developing these complications was shown to be significantly correlated with greater severity of DR [5]. Therefore, monitoring DR severity is crucial for the patient management and also an important end-point in several DR clinical trials; the FDA has recently approved the use of ranibizumab in the management of DR [6–8].

The Early Treatment Diabetic Retinopathy Study (ETDRS) stereoscopic 7-field (7F) imaging and grading protocol has been the standard of assessment of DR severity level and used in many DR studies and clinical trials [6, 7, 9–14]. Stereopsis is the perception of depth achieved by merging two slightly different images of the same location utilizing a stereoscopic viewer. In assessing DR severity, the perception of depth is generally presumed to: (1) help to differentiate neovascularization from intraretinal microvascular abnormalities (IRMA); (2) detect pre-retinal and vitreous hemorrhage; and (3) identify presence of macular edema. Despite these advantages, acquiring and grading stereoscopic 7F photographs are time-consuming, and highly dependent on the experiences of graders and training of photographers [15, 16]. Additionally, previous study showed that stereoscopic effect may not be critical for the assessment of DR severity [17].

In the recent years, one method developed for viewing the retina in a single shot, while retaining normal resolution of the original monoscopic photographs, is to create a montage by stitching monoscopic photographs together. Many publications in the literature applied montage to describe retinal diseases [18–21]. In a previous study, Li et al. compared assessment of DR severity using a monoscopic auto-mosaic image to standard stereoscopic 7F photographs [22]. In comparing to the montage, the mosaic is created from 9 monoscopic fields, one centered in the macula and others surrounding the macula. Meanwhile, the montage is created from 7 monoscopic fields [23, 24]. To the best of our knowledge, no one has applied the use of montage image in the assessment of DR severity and compared it to stereoscopic 7F images. Therefore, in this study, we want to compare the classification of ETDRS DR severity between stereoscopic 7F and non-stereoscopic montage of monoscopic 7F photographs.

## Methods

The study was conducted in compliance with the Declaration of Helsinki, the US Code of Federal Regulations Title-21, and the Harmonized Tripartite Guidelines for Good Clinical Practice (1996). De-identified images from the Diabetic Retinopathy Repository at the Ocular Imaging Research and Reading Center (OIRRC, Sunnyvale, California) were used for the analysis. Images were from subjects participating in an IRB approved DME clinical trial were utilized for this analysis. Clinical trials used standardized imaging protocol from OIRRC to capture images, and all patients were dilated. Eyes with complications of the posterior pole other than DR, such as age-related macular degeneration (AMD) and posterior uveitis, were excluded from the study. Subjects with media opacities or small pupil size leading to limitations in visualizing the retina were excluded from the analysis to reduce bias in grading.

### ETDRS stereoscopic 7-field color fundus photographs

A total of 16 digital 35° photographs, seven non-simultaneous color fundus ETDRS stereoscopic 7F pairs and one pair of fundus reflex images, were taken using high-resolution camera. Subjects’ pupils were dilated before imaging session. All images were taken by centralized reading center-certified photographers.

### Montage images

**Fig. 1:**
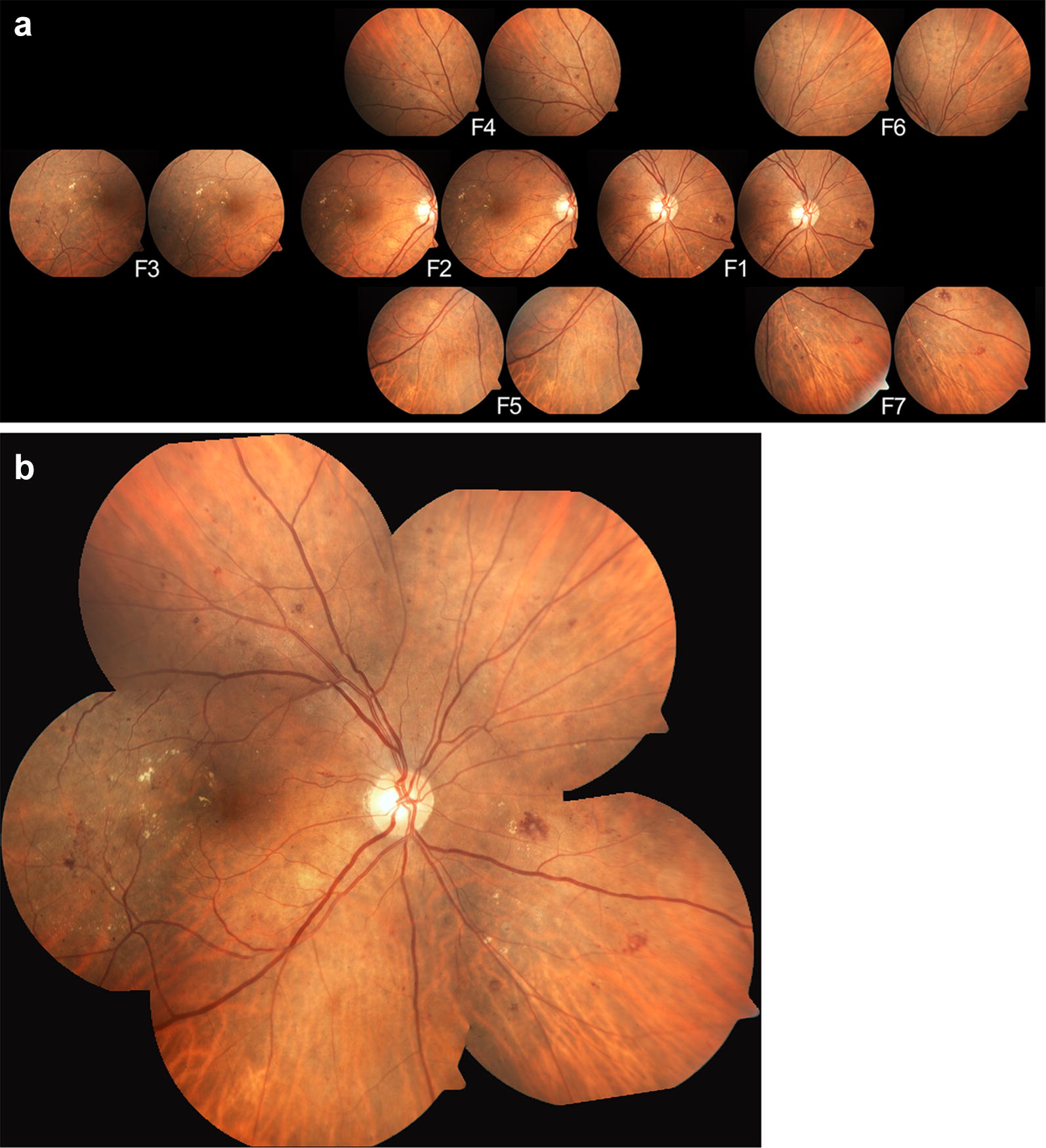
Example of standard stereoscopic 7-field photographs (**a**); and the corresponding montage image (**b**)

Montages were created manually by a trained technician using Adobe Photoshop CS6 Extended (Adobe Systems Incorporated, San Jose, CA). The better image of each stereoscopic pair from stereoscopic 7F photographs was chosen for montage assembly based on illumination, sharpness of blood vessels, and absence of vitreous artifacts. Images were adjusted and aligned manually following blood vessels and other characteristic such as retinal hemorrhages and hard exudates. The “Auto-Blend Layers” tool in the software was utilized to blend images into the montage. An example of ETDRS 7F stereoscopic photographs and the corresponding montage is shown in Fig. 1.

### Grading of images

All images were graded by three certified independent graders (MH, NN, and MSH) for assessment of DR severity based on DR severity scale adopted from *ETDRS Report 12* [23]. The graders had not participated in any examination of the subjects and were masked to all clinical information about the subjects. All three graders were first asked to perform grading on stereoscopic 7F photographs. Graders then waited at least 14 days before grading the montage images. The purpose of this approach was to prevent recall bias. Stereoscopic 7F photographs and the corresponding montage of each eye were assigned to different code numbers by a fourth team member (SB). The sequence of eyes in the set of stereoscopic 7F photographs was ensured to be different from the set of montages. For grading of stereoscopic 7F images, a pair of stereoscopic images for each field was displayed side-by-side on a 4 K high-resolution monitor and viewed with a Berezin Pocket 3Dvu (Berezin Stereo Photography Products, Mission Viejo, CA) stereoscope viewer. To grade the montage, the image was viewed on the same monitor and zoomed into view each field at the graders’ suitable magnification. All graders’ assessments of DR severity on stereoscopic 7F photographs and on montages were recorded into a spreadsheet. DR severity level for each eye was adjudicated as the central tendency among three graders. Discrepancies among readers were adjudicated as follows: if two graders agreed, that level was accepted; if all graders differed in grading, the median level was accepted [22].

### Statistics

Diabetic retinopathy severity level agreement between stereoscopic 7F photographs and montage was cross-tabulated, and κ-value and weighted κ-value were calculated to quantify the level of agreement. The κ-value was interpreted according to guidelines adopted from Landis and Koch [25]: < 0.20, poor agreement; 0.21–0.40, fair agreement; 0.41–0.60, moderate agreement; 0.61–0.8, substantial agreement; and 0.81–1.00, perfect agreement. Weighted κ-value was utilized to account for the degree of disagreement. The Stuart-Maxwell test of marginal homogeneity was also performed to assess differences in the percentage of severity levels between montage and stereoscopic 7F photographs. Sensitivity, specificity, positive/negative predictive values (PPV/NPV), and positive/negative likelihood ratios (PLR/NLR) for montage grading method were calculated using the grading of DR severity on stereoscopic 7F photographs as reference. p < 0.05 was considered significant on all tests in the analysis. Statistical analyses were performed using Stata, version 14.2 (Stata Corp LLC, College Station, Texas, USA).

## Results

### Baseline characteristics

A total of 50 eyes, 32 right and 18 left, were included in the study. The median DR severity score was moderately severe NPDR (level 47) on both stereoscopic 7F and montage images. The distribution of DR severity level assessed by stereoscopic 7F photographs was level 10 (0 eye); level 14/15/20 (0 eye); level 35 (6 eyes); level 43 (11 eyes); level 47 (13 eyes); level 53 (7 eyes); and level ≥ 60 (13 eyes).

### Stereoscopic 7F and montage agreement of severity levels

DR severity agreement between 7F and montage was cross-tabulated in Table 1. There was a substantial agreement between stereoscopic 7F and montage (κ = 0.745, κ_weighted_ = 0.867, p < 0.0001) in the assessment of DR severity score. Of 50 eyes, 40 (80%) eyes showed complete agreement, and 100% of the cases had agreement within 1-step (Table 2). The difference in percentage of DR severity levels between stereoscopic 7F and montage was not statistically significant (p = 0.6151).

**Table 1:** ETDRS DR severity score assessed from grading 7-field stereoscopic photographs compared to montage images

	Montage							
	10	14, 15, 20	35	43	47	53	≥ 60	Total
Stereoscopic 7F photographs								
10								0
14, 15 and 20								0
35			*2*	4				6
43			2	*8*	1			11
47				1	*10*	2		13
53						*7*		7
≥ 60							*13*	13
Total	0	0	4	13	11	9	13	50

Level 10: DR Absent; 14 and 15: DR Questionable; 20: Microaneurysms Only; 35: Mild NPDR; 43: Moderate NPDR; 47: Moderately Severe NPDR; 53: Severe NPDR; ≥ 60: PDR

*NPDR* non-proliferative diabetic retinopathy, *PDR* proliferative diabetic retinopathy

Italic indicates complete agreement

**Table 2:** Level of agreement for the assessment of ETDRS DR severity score on stereoscopic 7F compared with montage

	Montage vs. 7-field stereoscopic
Complete agreement	80 (%)
Agreement within 1-step	100 (%)
κ-value	0.745 (p < 0.0001)
Weighted κ-value	0.867 (p < 0.0001)

### Comparison of stereoscopic 7F and montages at different severity levels

The agreement in DR severity assessment at different DR severity levels between stereoscopic 7F photography and montage was shown in Table 3. The rate of agreement between stereoscopic 7F and montages ranged from 0.88 to 1.00 at different severity levels with the lowest at level 35 (mild NPDR).

**Table 3:** Diabetic retinopathy severity level: stereoscopic 7F photographs compared with montage image

Retinopathy severity	Sensitivity	Specificity	PPV	NPV	PLR	NLR	Rate of agreement
Level 35	0.33	0.95	0.50	0.91	6.60	0.71	0.88
Level 43	0.73	0.87	0.62	0.92	5.62	0.31	0.91
Level 47	0.77	0.97	0.91	0.92	25.67	0.24	0.91
Level 53	1.00	0.95	0.78	1.00	20.00	0.00	0.95
Level ≥ 60	1.00	1.00	1.00	1.00	–	0.00	1.00

*PPV* positive predictive value, *NPV* negative predictive value, *PLR* positive likelihood ratio, *NLR* negative likelihood ratio

### Sensitivity, specificity, positive/negative predictive values of the montage grading method

Sensitivity, specificity, positive/negative predictive values, and positive/negative likelihood ratio for montage at different severity levels were shown in Table 3. In comparing montage with stereoscopic 7F photographs, the sensitivity ranged from 0.33 to 1.00 at different severity levels. The lowest sensitivity was at level 35 (mild NPDR). Specificity and NPV for montage were similar across all severity levels. PPV for montage ranged from 0.50 to 1.0, and the lowest PPV was at level 35. PLR for montage at level 47 (moderately severe NPDR) (25.67) was higher than other levels that can be explained by high specificity at this level. Because there was a complete agreement at level ≥ 60 (PDR) between stereoscopic 7F and montages, PLR at this level was not able to be calculated. NLR for montage at level 53 (severe NPDR) and level ≥ 60 was zero because sensitivity at these levels was equal to 1.

### Intergrader agreement

Intergrader agreement was similar on both stereoscopic 7F and montages. The intergrader κ and weighted κ-values were shown in Table 4. There was a moderate agreement (κ-value ranging from 0.4705 to 0.5803, p < 0.0001) between graders on both montage and stereoscopic 7F photographs. The weighted κ-value ranged from 0.6511 to 0.7472, p < 0.0001.

**Table 4:** Intergrader diabetic retinopathy severity level agreement on stereoscopic 7 fields and montages

	Stereoscopic 7F (n = 50)	Montage (n = 50)	ETDRS report 12
Complete agreement (%)			53^a^
Grader MH vs NN	64	58	
Grader MH vs MSH	64	58	
Grader NN vs MSH	64	68	
Agreement within one step (%)			88^a^
Grader MH vs NN	98	98	
Grader MH vs MSH	94	96	
Grader NN vs MSH	92	90	
Agreement within two steps (%)			
Grader MH vs NN	100	100	
Grader MH vs MSH	96	96	
Grader NN vs MSH	96	96	
κ-value			0.42^a^
Grader MH vs NN	0.4705 (p < 0.0001)	0.5403 (p < 0.0001)	
Grader MH vs MSH	0.4710 (p < 0.0001)	0.5434 (p < 0.0001)	
Grader NN vs MSH	0.5803 (p < 0.0001)	0.5452 (p < 0.0001)	
Weighted κ-value			0.65^a^
Grader MH vs NN	0.7153 (p < 0001)	0.7472 (p < 0.0001)	
Grader MH vs MSH	0.6511 (p < 0.0001)	0.6975 (p < 0.0001)	
Grader NN vs MSH	0.7153 (p < 0001)	0.6873 (p < 0.0001)	

^a^These intergrader agreement values were obtained from *ETDRS Report 12*

## Discussion

Assessing severity of DR is important for both patient management and outcome measure in DR clinical trials. The early treatment diabetic retinopathy severity stereoscopic 7F photography imaging and grading protocol has been a gold standard for assessment of DR severity level and used in many DR clinical trials [6, 7, 9–14, 23, 24]. In this study, we compared assessment of DR severity between stereoscopic 7F photographs and montage image.

The results of our study suggest that montage image is comparable to ETDRS stereoscopic 7F photographs for assessment of DR severity. Previously, *Li* et al. employed a similar three-grader system to compare monoscopic mosaic image to standard stereoscopic 7F photographs for grading DR severity [22]. In their study, there was a substantial agreement between the mosaic and stereoscopic 7F photographs (κ = 0.62, κ_weighted_ = 0.86) for grading DR severity. Similar findings were also found between montage and stereoscopic 7F photographs in our study (κ = 0.745, κ_weighted_ = 0.867). Similarly, they noted complete agreement between the graders in 66.9% of images and agreement within one-step in 97.4% of the cases. In contrast, we noted a higher level of complete agreement (80%) and agreement within one-step (100%). The differences may be due to several reasons. Even though the mosaic image covered a larger area than the corresponding 7F photographs, it did not include entirely 7F retinal area. Moreover, the auto-mosaic feature of the algorithm did not choose the better-quality view when assembling the composite image. On the other hand, the montage images used in our study was assembled manually by a trained technician using the better-quality image of each stereoscopic pair based on certain criteria (illumination, sharpness of blood vessels, and absence of vitreous artifacts). We also utilized the “Auto-Blend Layers” tool in Photoshop masked out underexposed area in the overlapping regions and yielded a smooth transition in the final composite montage image.

Several studies have compared ultra-widefield (UWF) image and monoscopic 7F photographs to stereoscopic 7F photographs in the assessment of DR severity level in the literature [15, 17, 26, 27]. Although the UWF images provide larger view of the retina, the stereoscopic 7F photographs have higher resolution than UWF images. Therefore, the 7F photographs, which have the same resolution as montage, provide advantages for identifying small lesions. Aiello et al. have demonstrated that UWF images have lower sensitivity in identifying certain retinopathy lesions compared to 7F photographs [27]. Moreover, the UWF images provide no real color images, but only two monochromatic red and green SLO scans, resulting in semirealistic fundus images [15]. UWF imaging equipment is also not readily available, and until the day UWF cameras become the norm, we will need to rely on conventional fundus photography to evaluate DR. Advantages and disadvantages of different imaging methods in assessing DR severity are summarized in Table 5.

**Table 5:** Advantages and disadvantages of different imaging methods for screening and assessment of DR severity

Imaging methods	Advantages	Disadvantages
Stereoscopic 7-field	Stereopsis for detecting DME, NVE, NVD, pre-retinal hemorrhages, and vitreous hemorrhages	Time-consuming process and requires highly training photographers for capturing images [15, 16] Requires stereo viewer while grading images in order to appreciate stereo effect 14 images are captured
Montage	Viewing 7F area in a single shot while maintaining original monoscopic images Less photographs are taken (7 total)	Time-consuming in constructing montage and requires highly training technician Lack of stereopsis
Monoscopic 7-field	Less photographs are taken (7 total)	Lack of stereopsis
Mosaic	Less photographs are taken (9 total)	Lack of stereopsis Uneven transition between adjacent fields [22] Does not entirely cover 7F area although covers larger retinal area [22]
Ultra-widefield	Only one photograph is taken Covers much larger retinal area Viewing retinal area in a single shot Great screening tool for the presence of DR [15, 26]	Lack of stereopsis Lower sensitivity in detecting certain retinopathy lesions [26, 27] Semirealistic fundus images [15]

We analyzed the sensitivity, specificity, PPV, NPV, PLR, and NLR for montage grading methodology using stereoscopic 7F grading as a standard (Table 3). The montage grading methodology was found to be highly specific at all DR severity levels with a very high negative predictive value. However, there was a variation in terms of sensitivity of this grading methodology. The sensitivity of the methodology was lower at level 35 (31%) but significantly increased to > 70% at level 43 and 47 and reached 100% at ≥ Level 53 and above. The stereoscopic 7F photographs have a certain degree of overlap between the adjacent fields. Therefore, some lesions are usually seen in multiple fields. The advantage of such approach is that graders can use different views of same lesion to confirm their findings. However, the disadvantage is that the same lesion on multiple fields can potentially be counted as two different occurrences and give rise to a different severity score. The monoscopic montage image, on the other hand, decreases the chances of counting a single lesion twice since the entire 7 field area is visible together. Even though the “Auto-Blend Tool” allows a smooth evenly exposed image, it sometimes may result in over or under enhancement of an area. These differences in the montage grading and stereoscopic 7F grading methodologies can potentially explain the variation in sensitivities that we noted in our study.

The intergrader agreement for assessment of DR severity based on both montage and stereoscopic 7F imaging methodology in or study was comparable to other studies including the *ETDRS Report 12* (Table 4) [17, 22, 23]. In the *ETDRS Report 12,* complete agreement between graders occurred 53% of the time, and the κ-value was 0.42 [23]. In this study, complete agreement occurred on 64 ± 0% of stereoscopic 7F images, and 61 ± 5.8% of montage, and the average κ-value was 0.51 and 0.54 on stereoscopic 7F and montage, respectively.

**Fig. 2:**
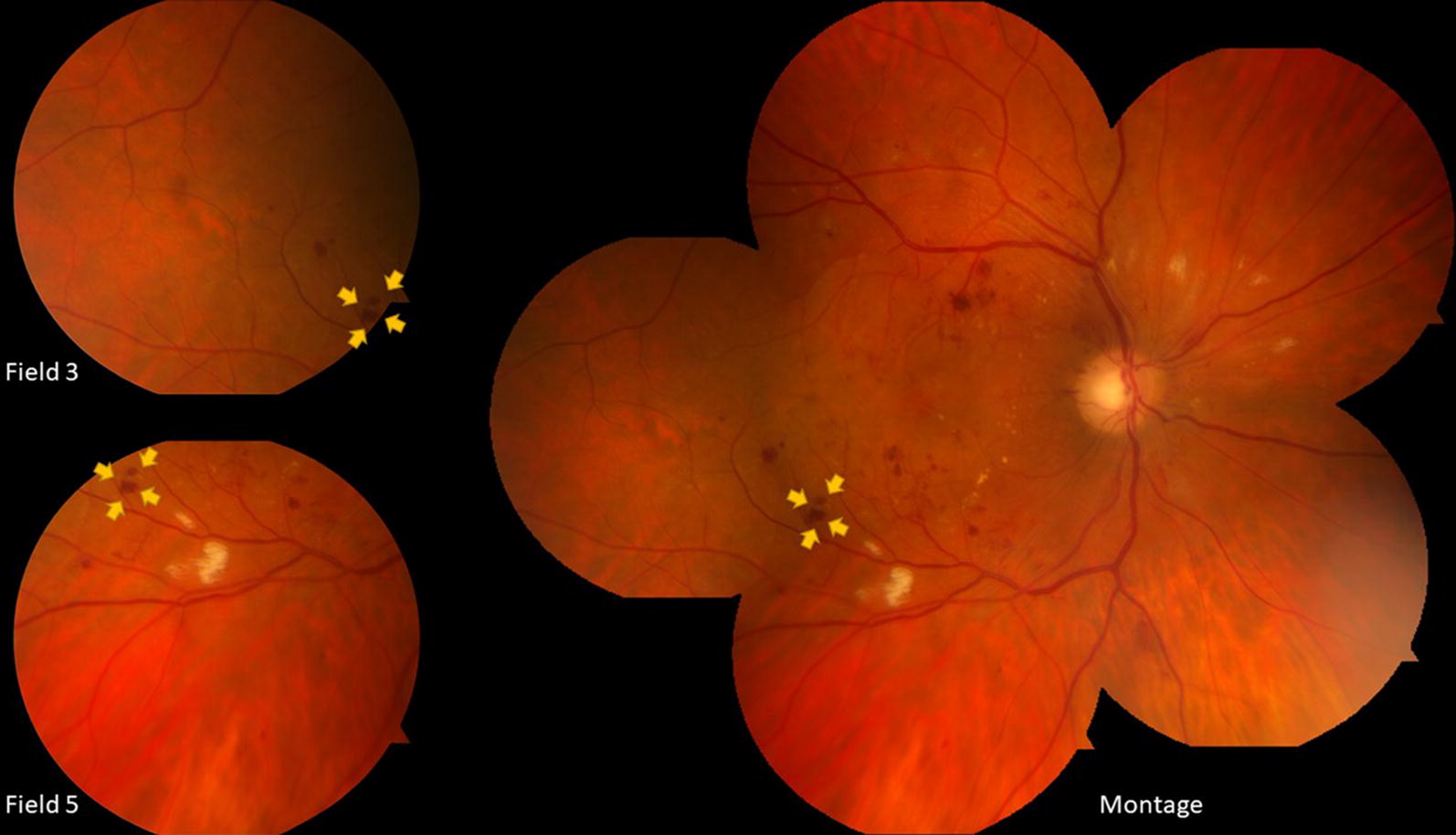
The retinal hemorrhage (yellow arrows) in Field 3 and Field 5 can be mistakenly counted as two occurrences. Meanwhile, this retinal hemorrhage is counted as only one occurrence on the montage

Considering recent developments of the artificial intelligence algorithms for detection and diagnosis of DR characteristics, montage images may have the advantage of allowing better and more efficient lesion quantification by these algorithms, especially in terms of decreasing the chances of counting same lesion as two occurrences (Fig. 2).

While montage grading methodology appears to be comparable to stereoscopic 7F photographs in assessing DR severity level, our study does have its limitations. The study had a small sample size, and variability of the subjects did not cover the entire spectrum of EDTRS DR severity scale. There was low frequency of PDR lesions (NVD, fibrous proliferations on the disc, and VH), and a small number of subjects with DR severity equal or less than level 35. In addition, construction of the montage is still a time-consuming process, and the technician was required to complete an intensive training process to be certified for montage construction. Another disadvantage of montage image is that due to its lack of stereopsis, the montage is less likely to provide the ability to detect and grade diabetic macular edema (DME) as compared to stereoscopic 7F photographs. However, presence or absence of DME does not impact the DR severity level and its assessment was not included in this study.

## Conclusions

In conclusion, we have found a substantial agreement in assessing ETDRS DR score on montage and stereoscopic 7F photographs. Intergrader agreement was also comparable in this study compared to other studies. Therefore, montage of the 7 fields can be used confidently as a possible and time-saving alternative imaging method to stereoscopic 7F photographs in assessing DR severity level in clinical research.

## Data Availability

The datasets used during the current study are available from the corresponding author on request.

## Abbreviations

DR: Diabetic retinopathy
ETDRS: Early Treatment Diabetic Retinopathy Study
IRMA: intraretinal microvascular abnormalities
AMD: age-related macular degeneration
PLR: positive likelihood ratio
NLR: negative likelihood ratio
PPV: positive predictive value
NPV: negative predictive value
NPDR: non-proliferative diabetic retinopathy
PDR: proliferative diabetic retinopathy
NVD: new vessels in disc
